# Supplementary material for: A Novel Role of Medicago truncatula KNAT3/4/5-like Class 2 KNOX Transcription Factors in Drought Stress Tolerance
Source: Int J Mol Sci. 2023 Aug 11;24(16):12668. doi: 10.3390/ijms241612668 (PMC10454132; doi:10.3390/ijms241612668)
Supplement: Supplementary file 1 [file ijms-24-12668-s001.zip › Figure_S2_Binding_sites_MtPDH_gene.pdf]

Figure S2

Analysis of the *MtPDH* (Medtr7g020820) promoter (Medicago truncatula Mt4.0v1, Phytozome genome ID: 285, NCBI taxonomy ID: 3880) for KNOX/BLH binding sites as defined in Bencivenga et al. 2016 [45]. Sequences highlighted in yellow represent the KNOX/BLH_bs1 (KGACM), those highlighted in bright green KNOX/BLH_bs2 (TGAYTGA), and those highlighted in bright light blue KNOX/BLH_bs3 (TGATKKGA). Phytozome key: 5'UTR CDS 3'UTR . Promoter analysis was done using RSAT Plants (https://rsat.eead.csic.es/plants/).

>M.truncatula Mt4.0v1|Medtr7g020820|chr7:6488218..6491368
forward|upstream=3000|downstream=0
GTTGGACCTTGTTTTTAGGGTTAAATTATCGTATCCTTTAAATCACCGTAAGTATATTTAGGGTTAAATTATTGTATCCA
TTAAATCACCGTAAGTATACAGTTGGATCACCTTTAAAGCAAGCAAATGTGTTTGTACATAAGGAAAAGTTATTCCCA
CTTGATTTTGACTAAAAACCCAATCTTCACAATTTTAAACTAAAGATGAAGTTGTTTTTAGTTATAGGTTTGTGTTTT
GATGATTTTCATTAGCTAGGTTTATTTGATGATGTGTTTATGTTGATTAGTTTTAACTTGGAATCATTTTAAAGCATCTA
GTAATCTTTGCCTCATTGTGTCATGTGATTCCTAGATGTCTTGTGTGTTGTTATGTACAAAAAATTTCATTTTTGAGCG
ACAACTGACAAAATCCTTCTTTGAGATGCTTGATTTGATTCATGGTTCATTGATTCATATCAAGCATTCTTTCTGATAT
TTTTCCTTATGTCTTACACCTTTTGATTCGATTCAAGATTTCATGAATCATATCACCAGTGAATCGATTCAAACTATCTT
GAATCGTATCTTTGGTTCTGTCTGGACCTTGTTTTTGCATGTGTGAATCGATTCAGTATTTGATGATTCGTATCAATCT
TCATCTGAGTTCATTTGAATCGATTCATATATATGTTGATTTGTATCATTCTTCATTATTCAGATTGCCTATTTTCAGCAG
TCTGAATCGTTAGTGAATTTAGTCACTTAGTTGTTTTGGGGCAAATTTTTGCACTTTCCACTATTTTCTATATATACTCA
TATCCCCGTAACACCCAAACCACTTAATCATTTGCATCTATTCTCTCTAAGTCTCCAAACCTCAATTTCCATTTATTCTTC
AGATTAAACACTTTTTCATTGATTGGAAATCAATGATTTAAAGTGAATTGAGTAATTTCATTCCACCTTCAAGGGTAT
TGGAGGTTCTTCCTAACCTTCTTGAGTTGAAGGGAATCCAACCACCGAACAGAGCAAAGAAGAGTTCAAGGGAA
GAGCTTCAAGGATTGCACGAGTGACTGAGGATTCGAAGACTTGAAGAAACACTAATTTCAAAGGTATTTGAAGAT
TGGGGGAGTGATTGCGTAGAAATTGAAAATTAGGGTAATTGAGGAAATTCTTGTGATCACAACTTTATAATCAAACA
TTCAATTTTAGTGAAAGTCCTCGATATTAATGTAACATGAGTGATGACTGAACTATTAAGTTGCTATAGCTTACCTTGT
GTTAGAGAATCTTTGTTTAGTTTTTAATAAAATTGATTTAGCTTCTTACAAAAAGATACTAATATTATACTGTGTTTTTT
ACTTTTCATATGGCAAGATAAAATTGTATCTCCCCATTTTCTTTTTTTAAGTATTAATCGCTTATTTTACATATATTAACA
AAAGTCACTTACGTAATTTATGTGTTTGTTTTTGCACACTCTGATTATCAAAATTACATCTTGTGTATTTTATGTATTCA
ATATTGATTTTACATATTAAAAGAAAAATTAGTATTATTAATCAGGATTGTCAAGAATAAATAATTTGCACTTAGTAATT
TTTTGTCATCTGAATTGAAATGGAAAATAAACATCACAGTGACATTACCAAACGACAACACTTAGATTCAAACTCTA
ATATCGGTCGTGCCACTAAAGATATTGTAGTAATTTTAACAAGTTATAAACTTTTTTTTTCTTTCTTAAGTGTAGTTTTG
CCCTCCCTATTTTGACTCAAACGGAGTTTTACCCCCCTATTTTATAACTCGGAATTTCACCCCTTTGTTTTACATTTTCC
AGGATTTTGCCCCAAGTCAAATTTAAATTCATTTATGTTGATGTGGCATTGCTATGTGAATTTTAAAATATTATCTTATT
ATTCACCTAATTAATTATTTAAAATAATGTAAAAAAAATCATTAATCATTTAAATTAATTTTTTAATTACAAAATTTGGAA
TAACTTTTTTAAAAAATACCATAAAATCCAGAAAAAAGCAGGAATTATAAAACAAAATTATAAAACCGGGAGGAGA
AAAAAGAAGTAAAAAAGCATAAAAAATACTGGAAAATTGAGATAAAAGGTAGGAAAAAAATCAGAATAAACAAAA
GAAAATCCCATTAAAAATACATGGAAGGAAATATAAGTAAAATGTTTTTATTTTTTGAGCAAAGTAAAATGATTTTTT
AGATTTTGGATTTTGGTGTGTCCGTATAAGCAAAATGATTTTACACCAAAAAAAAAAGATTTTTTGAAGGAAATATT
AGCAAAATGATTATTATTATTATTTTAAGTTACATTTTTTTTTTATTGATTTTTTTCTTTTTTTTTTTAATTCAGAATATTT
AATATAAATAATTTAAATTAGTGGATACTTTTTTAATATTATTTAAGCCACATCAGCACCGCCACATAAGCCAAATCCTG
AAAAATATTAAATTGGGGGGCAAAACTCCGAAAAACATTAAACTGGGGGGGTGAAATTCGGAGTTTTAAAATAGA

GGACGTAAAACTCCGTTTGAGTCAAAATAGGGGGGCAAAACTGCACTTAAGCCATTTTTTTTTAAATGGAATGATT
AATACCATATGAAACTTACGTACAATTTTGATATTTAAAGATGTCCAAATTGGCAATATTTGATTATTTTTTTTTACAGA
TGGCAATATTTGAATTGTTAACAGTTTTTTTCTTAATGGTGTTTTTTTAATAGTGTCTATAATTAGGAAAATGAAGAAA
TAGGGAGGTACTTAAGAGTAAACAATTTAGTCAAAGAGAGATAAAAAGAATCTATCAACATGAGTTGTAGTGTTACT
ATAGTGAGTAAGTTCCAACACATGATGAGTGTATCCGCGTAGATTCTGTGTCAGCATTTGAGTACGTTCGTACCAATC
TCAAATCTATCTTGAATCTTCAATTTCTTAGCTGTGTTTGTTTTGTTTGTTGCAACAACTTACCACATGGTACTCTTAG
CTACCATCCATTTTTACAACACAATTTACTCATATTAAACTAACTTACTCCAATAATTTAGGAAACATGACTAAAACAAT
TTTCTAAATTAAACCAACTTTGGTCAAAAACTATAACAAACTTACTCATATCCCCACCAACCAATTCACACATCATCCA
CTTGACTCTTTTTATCTTTTTTAATCACACCCTCCTTTCTTTTAGCTACCACATTCTTCTTTTCACCCCTATTTATACATCT
CCTTCTAATTCATTCTTACTTCACAAGCATACACATTTTGTTGTATCTCATTACATTAACAAAAGTATCTGGTTTTTTATT
CATCATATCAAAACATATATCTTCTTGTTTATATCATATAAATCATCTTTGACCGGTTGATGGCCACCAGAGTTATCCCA
CCAAGAATCCTAAGGAAACTCCGTTACAACACTGCCACAAAACCGTTCCAACCTGCCCTCACCTCGCCCGCTGTCG
CTCCTGCGTCTAATATTTTAGACCAAAAACTGCCATCATCGGTAACAACCATCCTCCCTCCCGACGCCAATCTCGACT
TCCACGACGTCGAAAAACTCTTTTCGCACGTCCCAACCAAAAAACTTCTCAAATCAACTGCTGTCCTCCATGCTACC
GCGGTCGAGCCAATGGTTGACCTCGGTACATGGTTGTTGAGGTCTGATCTCATGCAGACCAATAATCCATTAAGTAA
CCTTGCTATGGCTACCACACGTGCCACTTTTTTCGATCATTTTTGTGCCGGAGAAGATGCTATCACCGCTGGAAAAA
GTATTGCCGGGTTGAATGAAGCAGGTTTACGTGGAATGCTGGTTTATGGCGTTGAAGATGCTCATGATAATGCTGG
CTGTGATCGCAATCTCAAAGGTTTTCTTCACACCGTTGATGTCAGCAGATCGCTTCCTCCATCTTCGGTAATCTCTTCT
TTATTTCATCTCTCTATTTCAAAATGTAACTGTTTTCTTTTTTTGCTTTTTTGGTTCGGTTTCTTCATTGTCACAGAAGA
GAGATGCAATCTGCTTTAATGGATCCAAATTCAATTGAGAATAACCGTTTTGATGGTGCTGAGTTCCGCATTCGTGA
CTGCAACCGCAATTTAATACAGCATTGATTGATGACGTTGTTGCTGCTGCCGTGATGTATCTGGTGTCCGTGTCTGAT
TCCGTGCTTCTGAGGAATGAAAAGAAAACTAAGCATGGAACATTGAAATTGGAAATGGAATTGGAATTTGATTCAT
GAAATTGATCAAAGCATGGAAAGGAACAATGGAATTGGAATGGAACATTGGAATTGGAACAAACAAACAAACAAT
TGTTTTTGGTAAAAGACTCAAGCACTAATAAGCCACACAGAAAAAACTGTGGTTGCTGGTGCATTTTTATATACTTT
TTTTAAAATTACTTAAAAAAAAGTGTTATGAATTTGAGGGGTTATTTTAATTTTAAATTTAACATTATGATTATGAATGT
TATGTACTAATATTTTCAAAATTGAACTTTTTTGTGTTATAGGTAAGCTTTGTGATTGTGAAGATTACTGCAATATGTCC
AATGAGTTTGCTTGAAAGAATGAGTGATTTGCTGAGATGGCAGAAGAAAGACCCTTCTTTTGTTTTACCATGGAAG
CAAGATTCATTGCCAATTTTCTCAGAGTCAAGTCCTTTGTACCATACAAGGAAGAGACCAGAGCCATTAACAGCAG
AAGAAGAGAGTGATCTTGATCTTGCTAACAAGAGATTCCTTGAGCTTTGTCAGAAATGTGTGCAAGCCAATATTCC
ATTATTGGTTGATGCAGAACATACTTCAGTTCAACCTGCTATTGATTACTTTACATACTCTTCAGCAATTTTGCATAAC
AAAGGTGAAAACCCTACTGTGTTTGGAACCATTCAAACTTATTTGAAAGACGCTAAGGAGAGAATGTTGTTGGCAT
CAAAGGCTGCTGAGAAAATGGGGATACCAATGGGATTTAAGTTGGTTAGAGGTGCTTATATGTCTAGTGAAAGAAA
ATTGGCTGCTGATTTGGGATATGCTTCACCAATTCATAACACTATTAAGGATACACATAAGTGTTTCAATGATTGTTCA
GATTACATGCTTGAGAAGATTGCTAATGGTCCTGGTGGAGTTGTTCTTGCAACTCATAACATTGAATCAGGTATATAT
TATATGATATTATGATGATAAGGAAATTAATGTATGCAATTGAGGGGTATGGTATGAGTTTGATTTAATTAATGTTGTTT
TTTGTATAGGAAAATTGGCTGCTGCAAAAGCACATGAATTAGGGATTGGAAAGGTGAACCATAAGATGGAATTTGC
ACAACTATATGGAATGTCTGAGGCACTATCCTTTGCTTTGAGCAATGCAGGGTTTCAAGTTAGCAAGTACATGCCAT
TTGGACCTGTTGAGACTGTTATGCCATACCTCTTGAGAAGGGCTGAGGAGAATAGAGGAGTGTTGGCTGCATCAG
GATTTGACAGGCAACTCATGAGGTAAAAAATTGAAAGAAAAAAAAATTAACCATTTCACTTTTACTCTAGTTAATTG
GTCAGTGTGTCGGTGTCCGACACCGGGACACGCTTAATCCGAGGAGTGTCCGTGCTTCATAGTTTGTGACTCATGA
AATGTAACATTTGGTTGATTCTGTTTCTGACATTGATTTTGTTTTGTGTGTGACAGGAAGGAGTTGGTCAGGAGAGT
AAAAGCAACTGTGCTTTGAATTTGTTGGATGAGTTGATGGGATGTAATAATGTAGGCAACAGGATTCACCATTCTTT
GTACAAATTAGAGAGAATCCTGTAATTTGCTTAAGTTTTGTGCATTCAAGTTAATGAAATGTGCATTTGTCTTGATGT
GTATTGATCCACATTTCCACTTGTTTGTACATTAATGCCATTGAATTTTTTAAGAAATTGTTATTCATAAAGTGTCTTTT
GTGTTTGAGGTGATTCCATTTGAATTTGGTTAGAAAACTACAAATGCAAAAAAGCAAATGAAAATAATAAGATAGC
AACTTGTGCAATACAAAAAGGTAAATGGAATATGGAAATAATTTAAGGTAGCAACTTGATCTTGAATTTGGCTATGA
TTGGTAGCCATCATTTCACTATACTATAGCATACAAATGTTGAAAGAGATCAAT
