# Supplementary figures and images for: A Novel Role of Medicago truncatula KNAT3/4/5-like Class 2 KNOX Transcription Factors in Drought Stress Tolerance
Source: Int J Mol Sci. 2023 Aug 11;24(16):12668. doi: 10.3390/ijms241612668 (PMC10454132; doi:10.3390/ijms241612668)

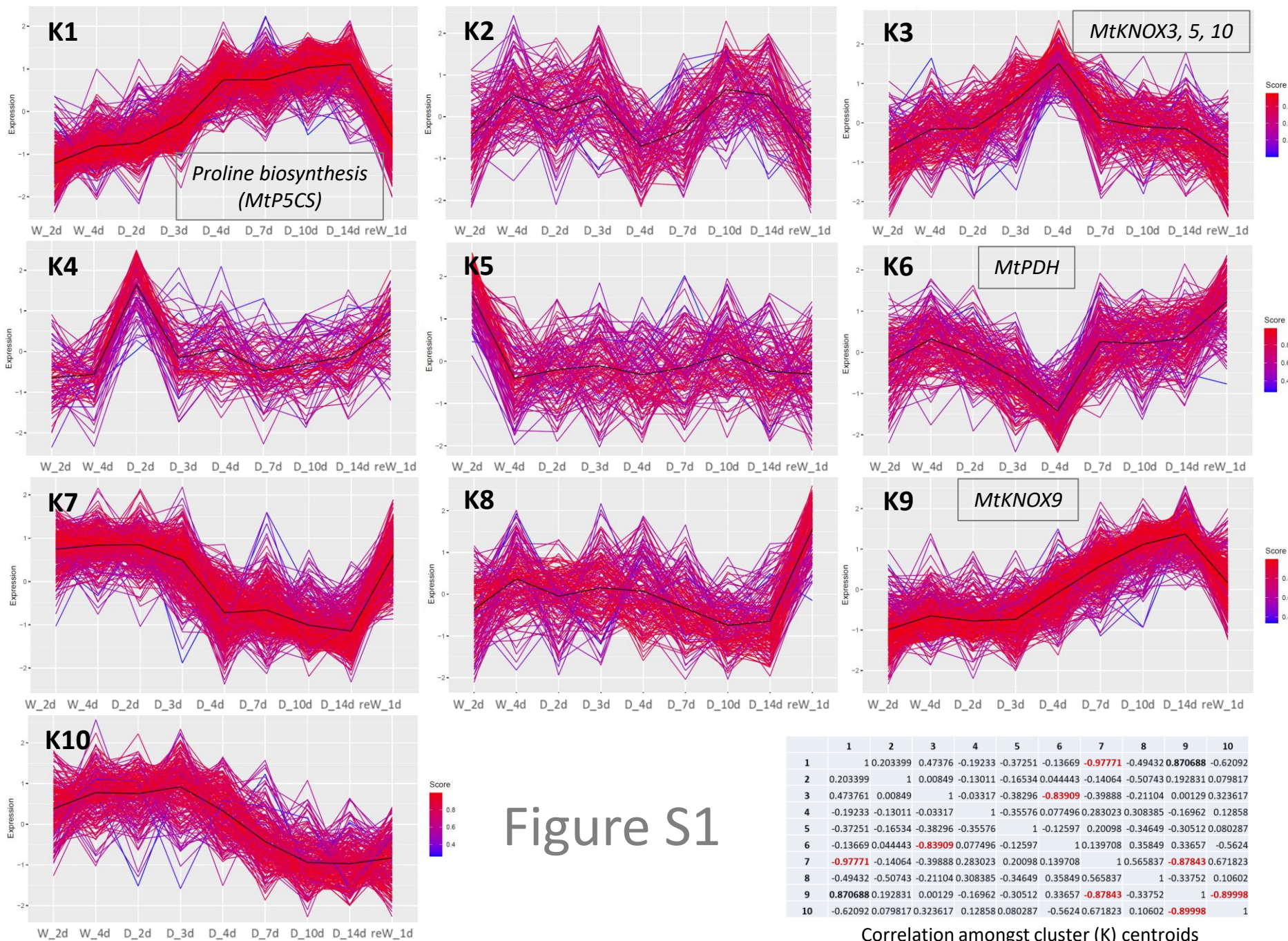

Supplement: Supplementary file 1 [file ijms-24-12668-s001.zip › Figure_S1_K-means cluster analysis of microarray metadata.pdf]
